# Mammary Duct Ectasia Presenting As MRI Non-mass Enhancement in a RAD51C Carrier With Pituitary Adenoma

**DOI:** 10.7759/cureus.106937

**Published:** 2026-04-13

**Authors:** Komal Ijaz, Wei T Yang, Erika Resetkova

**Affiliations:** 1 Pathology, MD Anderson Cancer Center, Houston, USA; 2 Radiology, MD Anderson Cancer Center, Houston, USA

**Keywords:** breast, duct ectasia, hyperprolactinemia, mri non-mass enhancement, pathology case report, rad51c

## Abstract

Mammary duct ectasia is a benign breast condition that can mimic malignancy on MRI. We describe a woman with a pathogenic RAD51C variant who entered high-risk MRI surveillance at age 53 and was found to have segmental non-mass enhancement in the central left breast. MRI-guided core biopsy demonstrated duct ectasia with inspissated eosinophilic secretions, dense periductal lymphoplasmacytic inflammation with concentric fibrosis, and coarse microcalcifications without epithelial atypia or carcinoma. Her history included hyperprolactinemia from a growth hormone- and prolactin-positive pituitary adenoma diagnosed at age 41 and treated surgically, raising the possibility of an endocrine contribution to ductal dilatation and secretory stasis. This case emphasizes the importance of radiologic-pathologic correlation to avoid overdiagnosis while highlighting a potential link between long-standing hyperprolactinemia and mammary duct ectasia in susceptible patients.

## Introduction

Mammary duct ectasia is a benign condition characterized by dilatation of lactiferous ducts, often associated with periductal inflammation and fibrosis [1-3]. It involves extralobular ducts and shows varying degrees of periductal inflammation, periductal fibrosis, and duct dilatation. Foamy histiocytes may be present within luminal secretions and can infiltrate duct walls [3]. While most cases are described in peri- and postmenopausal women, and rarely in children [4], ectasia is increasingly detected incidentally in younger patients on advanced breast imaging [5,6]. With widespread breast MRI, duct ectasia may sometimes be mistaken for intraductal neoplasia [7,8].

The etiology is multifactorial. Hormonal influences, particularly prolactin, may contribute to ductal changes [9,10]. Hyperprolactinemia, often due to pituitary adenoma, can produce breast-related manifestations, but structural ductal changes detectable on imaging are rarely reported [11,12].

We present a case of mammary duct ectasia detected on breast MRI in a patient with hyperprolactinemia due to a pituitary adenoma, highlighting a potential endocrine contribution [10]. This rare case underscores the need for clinical and endocrine correlation when evaluating ductal abnormalities [1,11]. Accurate recognition of mammary duct ectasia is clinically important, as misinterpretation, particularly on imaging, may lead to unnecessary biopsies, patient anxiety, or overtreatment.

## Case presentation

At the age of 41, the patient presented with a five-year history of progressive fatigue, dental spacing, snoring, headaches, visual pressure, polyuria, hyperhidrosis, and acral enlargement. She reported irregular menses, impaired fasting glucose, and depressive symptoms.

Physical examination revealed features of acromegaly, including frontal bossing, prognathism, widened dental spacing, acral enlargement, lax soft tissues, and diaphoretic skin with acanthosis nigricans. Pituitary MRI showed an 8-mm left-sided adenoma displacing the gland to the right without optic nerve compression. Laboratory evaluation demonstrated increased growth hormone (GH) and elevated insulin-like growth factor 1 (IGF-1). The patient underwent transsphenoidal resection at an outside institution; histopathology confirmed a pituitary adenoma, which was positive for GH and prolactin. Postoperatively, symptoms resolved with normalization of IGF-1, and she remained disease-free on follow-up.

**Figure 1:**
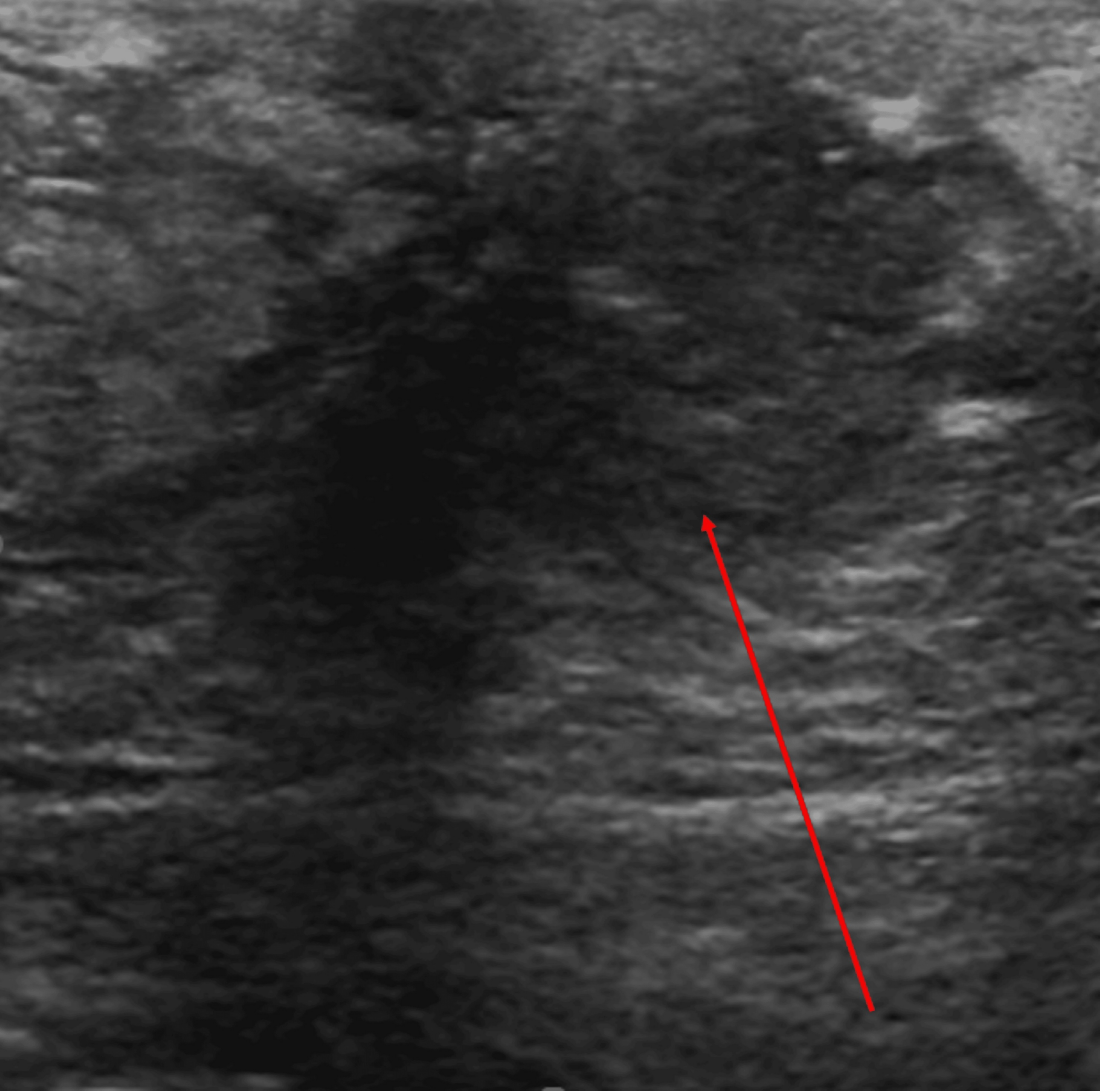
Targeted breast ultrasound showed a 6 × 4 millimeter smoothly marginated complex cystic lesion in the right breast at the 11 o'clock position (indicated by arrow).

At age 46, the patient presented with a two-month history of a right breast lump prompting further diagnostic evaluation. Ultrasound demonstrated a 6 × 4 mm smoothly marginated complex cystic lesion at the 11 o'clock position, Breast Imaging Reporting and Data System (BI-RAD) category 4 (Figure 1). Fine-needle aspiration showed acute inflammation and granulation tissue without malignancy. She experienced recurrent cyst inflammation requiring aspiration and antibiotics, with negative cultures.

Family history included ovarian cancer in her mother, ductal carcinoma in situ in her sister, and a maternal half-first cousin with a reported breast cancer gene (BRCA1/2) pathogenic variant. Genetic testing revealed a RAD51C c.404+2T>C pathogenic variant. She was enrolled in high-risk breast MRI surveillance at age 53.

Bilateral breast MRI was performed on a 3-T system following administration of gadobutrol. Sequences included axial T1-weighted pre- and dynamic post-contrast series with subtraction, axial T2-weighted, and diffusion-weighted imaging with apparent diffusion coefficient (ADC) mapping. Kinetic analysis was performed on a dedicated workstation.

**Figure 2:**
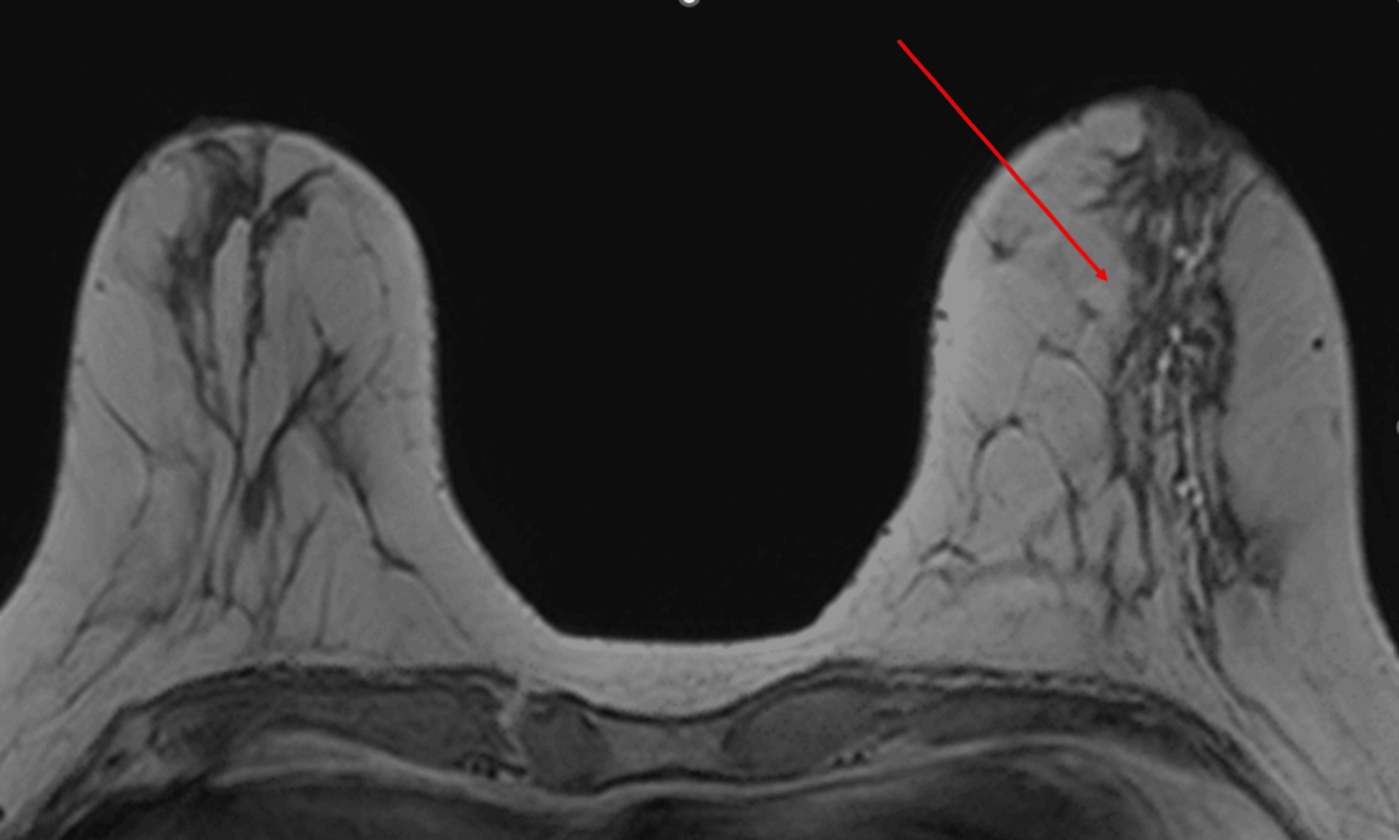
Breast MRI axial T1 pre-contrast: T1-hyperintense ductal ectasia in the central left breast (indicated by arrow).

**Figure 3:**
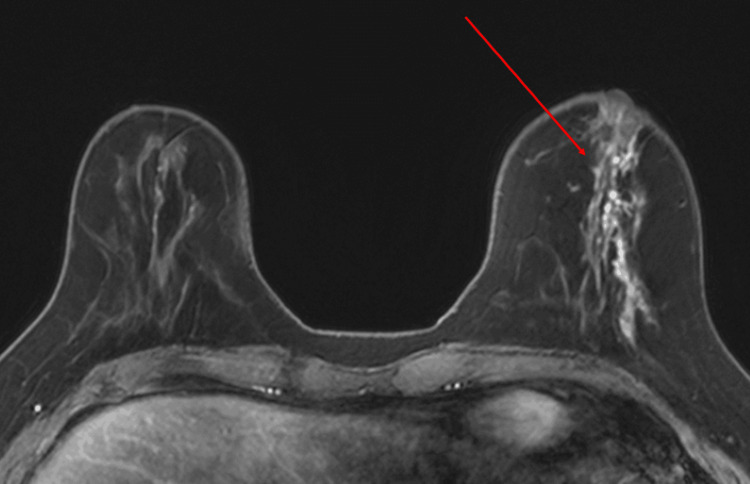
Breast MRI post-contrast dynamic: segmental non-mass enhancement with plateau kinetics in the central left breast (indicated by arrow).

Axial T1-weighted pre-contrast images demonstrated T1-hyperintense ductal ectasia (Figure 2), and dynamic post-contrast images showed segmental non-mass enhancement with plateau kinetics in the central left breast (Figure 3). “Non-mass enhancement” on breast MRI refers to areas of abnormal contrast uptake that do not form a discrete mass but instead appear as diffuse or regional enhancement, which can be difficult to distinguish from malignant processes. Differential considerations included fibrocystic change, pseudoangiomatous stromal hyperplasia, ductal carcinoma in situ, and invasive carcinoma.

**Figure 4:**
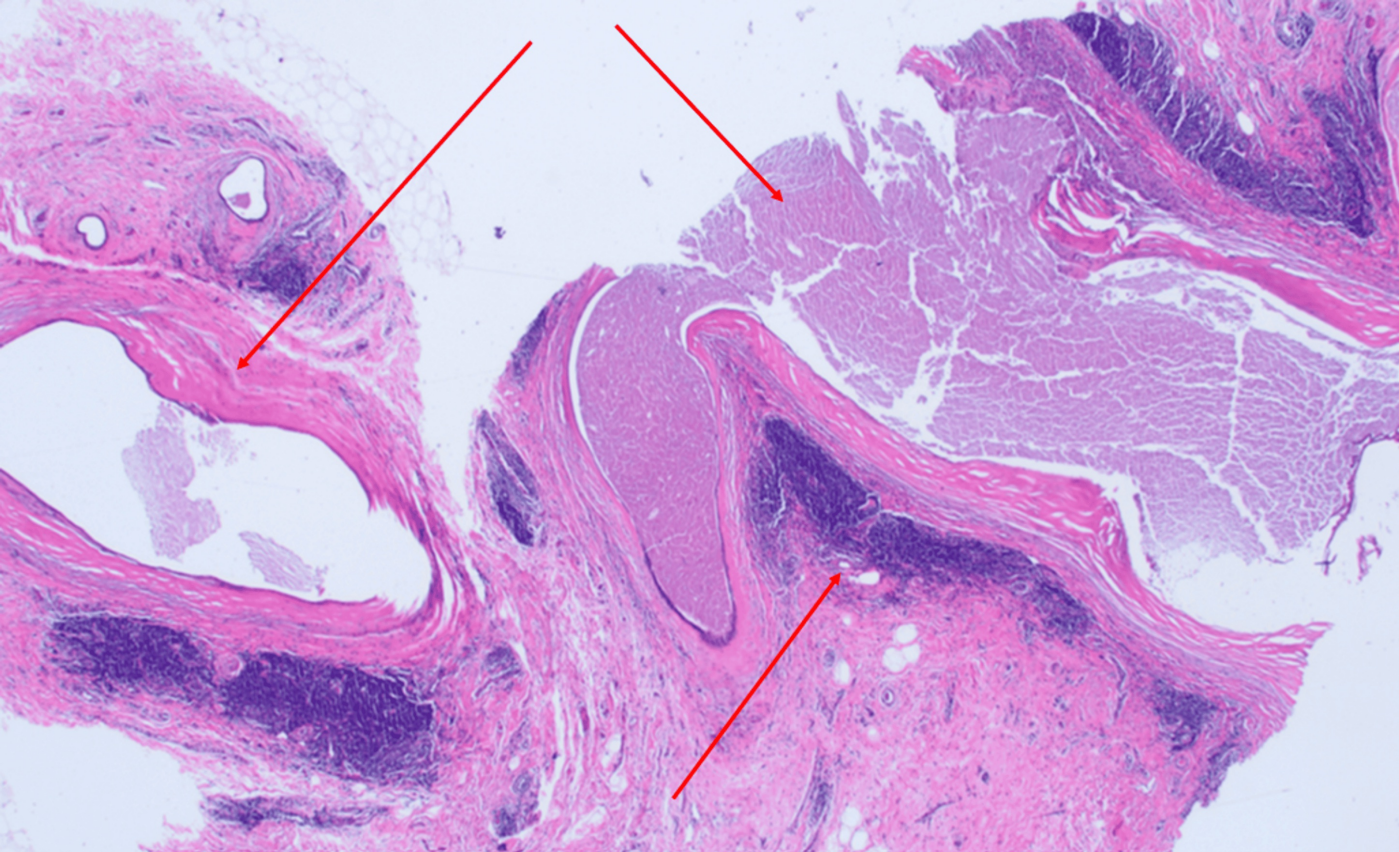
H&E-stained section, magnification 10x. Irregularly dilated ducts containing inspissated eosinophilic secretions, dense periductal lymphoplasmacytic inflammation, and concentric fibrosis (indicated by arrows).

**Figure 5:**
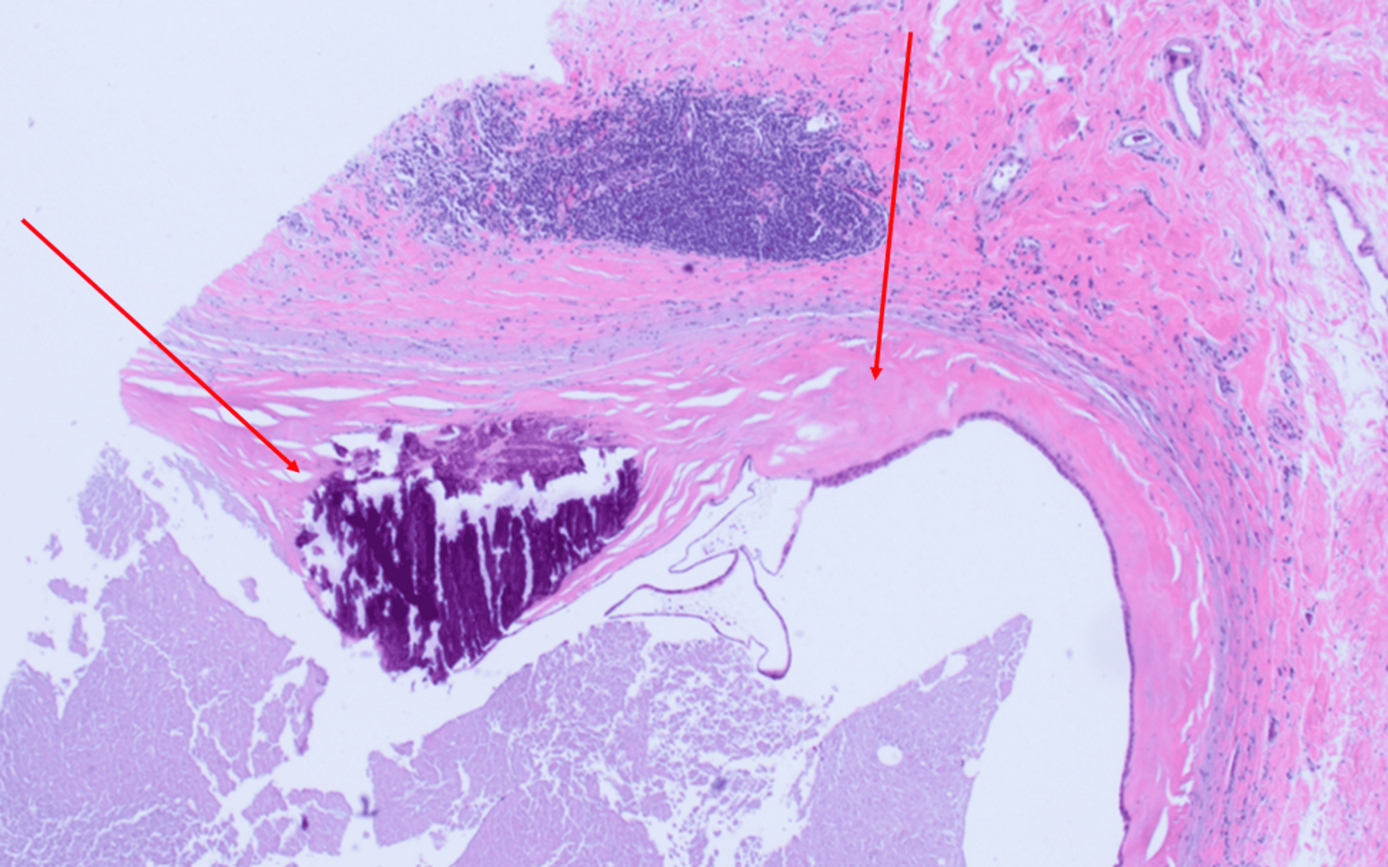
H&E-stained section, magnification 20x. One coarse dystrophic microcalcification is located within the thickened fibrotic duct wall (indicated by arrows).

MRI-guided core biopsy was performed from the central left breast. Pathology examination shows multiple irregularly dilated large ducts containing inspissated eosinophilic secretions (Figure 4). Dense periductal lymphoplasmacytic inflammation with concentric fibrosis was noted. Coarse dystrophic microcalcifications were present, predominantly within duct walls (Figure 5). No epithelial atypia, necrosis, or carcinoma was identified; the myoepithelial layer was intact. Immunohistochemistry was not performed; routine H&E was sufficient for definitive diagnosis.

Pathology final diagnosis

Mammary duct ectasia, benign, with dilated ducts, inspissated secretions, periductal stromal fibrosis, chronic lymphoplasmacytic inflammation, and dystrophic microcalcifications, negative for malignancy.

Differential diagnosis

Duct ectasia should be distinguished from granulomatous mastitis, subareolar abscess/duct fistula, lobulocentric fibrocystic change, and ductal carcinoma in situ or invasive carcinoma. Key distinguishing features include involvement of large extralobular ducts, intact myoepithelium, periductal lymphoplasmacytic inflammation, concentric periductal fibrosis, inspissated luminal debris, and elastic fibers within the duct wall.

## Discussion

This case illustrates the classic histopathologic features of mammary duct ectasia identified in the setting of MRI-based high-risk breast surveillance. Clinically, duct ectasia may present with nipple discharge, nipple inversion, or a periareolar mass and is well known to mimic carcinoma on imaging studies, particularly in the subareolar region [1,3,11,13]. Histologically, duct ectasia is characterized by irregular dilatation of large extralobular ducts accompanied by periductal fibrosis and a chronic inflammatory infiltrate composed predominantly of lymphocytes and plasma cells. Eosinophilic, inspissated luminal secretions are common; foamy macrophages may be present, and coarse dystrophic calcifications can develop within the surrounding fibrotic stroma [1-3,13]. Recognition of these features is essential to prevent misinterpretation as malignancy and to avoid unnecessary surgical or oncologic intervention.

Radiologically, the MRI findings in this case closely paralleled the underlying pathology. The pattern of segmental non-mass enhancement with ductal morphology and plateau kinetics corresponded to dilated ducts filled with secretory material and surrounded by chronic inflammation. Prior studies have demonstrated that benign ductal processes, including duct ectasia, can produce suspicious MRI patterns that overlap with those of ductal carcinoma in situ or invasive carcinoma [5-7]. This case underscores the importance of integrating BI-RADS descriptors, enhancement distribution, and kinetic curves with histologic correlation to avoid misclassification as carcinoma [6,7]. The patient is doing well and remains on routine follow‑up.

The patient's history of a pituitary adenoma with growth hormone positivity and focal prolactin expression, followed by postoperative endocrine remission, raises the possibility of an endocrine contribution to the development of duct ectasia. Historical observations have suggested an association between pituitary disease, hyperprolactinemia, and mammary duct ectasia [10]. Although not many patients with elevated prolactin levels develop duct ectasia, chronic prolactin excess provides a biologically possible substrate for ductal secretory stasis, particularly in non-pregnant, non-lactating individuals. Prolactin is the principal hormone responsible for secretory activation in the mammary gland, and in the absence of coordinated milk ejection, persistent low-grade secretory activity may lead to progressive accumulation of thick, protein-rich material within major lactiferous ducts. As intraductal secretions accumulate, intraluminal pressure increases, predisposing large ducts, especially those in the subareolar region, to progressive dilatation. With continued distention, the ductal epithelium may become attenuated and more susceptible to mechanical injury, facilitating leakage of luminal contents into the adjacent stroma. Duct ectasia does not arise from secretory overload alone; rather, the characteristic lesion develops when ductal injury initiates a chronic inflammatory response, tissue reaction to extravasated secretions, and subsequent periductal fibrosis [1,11,13]. In this patient, the combination of marked ductal dilatation, abundant inspissated secretory debris, and a prominent lymphoplasmacytic infiltrate supports a model in which prolactin-driven secretory stasis set the stage for advanced ectatic change.

Despite this biologic plausibility, most patients with prolactinomas or hyperprolactinemia do not develop clinically significant duct ectasia. Several factors may account for this observation, including early initiation of dopamine-agonist therapy with normalization of prolactin levels, inter-individual variability in ductal responsiveness to prolactin, preexisting microscopic ductal degeneration or impaired drainage acting as local cofactors, and relative resistance of the mature or involuting breast to pathologic secretory overdistention [9,11,12]. The occurrence of duct ectasia in pediatric patients further supports the concept that factors beyond age-related involution and parity, such as hormonal influences, ductal anatomy, or impaired ductal drainage, may contribute to disease development in susceptible individuals [4]. Although prolactin excess is a systemic hormonal influence, duct ectasia often presents unilaterally rather than bilaterally. This asymmetry suggests that local ductal factors play a critical role in lesion development. Subtle preexisting differences in ductal anatomy, drainage efficiency, or focal ductal injury may render one breast more susceptible to secretory stasis and progressive dilatation than the contralateral side. Minor, clinically silent obstruction or age-related ductal degeneration affecting a subset of major ducts could preferentially amplify the effects of prolactin-driven secretory activity on one side. In this context, systemic hyperprolactinemia may act as a permissive or amplifying factor, while local anatomic or microenvironmental conditions determine laterality and focal disease expression. Accordingly, prolactin excess may create permissive conditions for secretory stasis, but additional local or structural factors likely determine progression to the full inflammatory-fibrotic phenotype of duct ectasia [10,13].

The differential diagnosis of duct ectasia includes several entities with overlapping clinical, radiologic, or histologic features. Granulomatous mastitis typically presents as a firm, often painful mass and is characterized histologically by lobulocentric granulomatous inflammation rather than involvement of large extralobular ducts; it lacks the inspissated luminal secretions and elastic fibers characteristic of duct ectasia [2,3]. Subareolar abscess and mammary duct fistula usually arise in the context of periductal infection and are frequently associated with squamous metaplasia of duct epithelium and acute neutrophilic inflammation, whereas duct ectasia shows predominantly chronic lymphoplasmacytic inflammation without abscess formation or fistulous tracts [1,11]. Duct ectasia is also distinct from lobulocentric fibrocystic change, which primarily involves terminal duct-lobular units and lacks the elastic fibers typically present within the walls of ectatic large ducts [1-3]. Finally, ductal carcinoma in situ and invasive carcinoma may mimic duct ectasia radiologically but are distinguished histologically by epithelial atypia, proliferative architecture, and loss of an intact myoepithelial layer-features absent in duct ectasia [1-3].

Radiologic-pathologic correlation is therefore critical. Non-mass enhancement in a segmental or subareolar distribution with ductal morphology on MRI, when interpreted in conjunction with benign histologic findings, supports a diagnosis of duct ectasia and refines the imaging differential diagnosis [5-7]. Such correlation helps prevent overdiagnosis and overtreatment.

Recognition of hyperprolactinemia-associated duct ectasia is clinically important, as this entity may clinically or radiologically mimic subareolar neoplasia, produce concerning nipple discharge, or, in severe cases, raise suspicion for inflammatory carcinoma. Understanding duct ectasia as a benign secondary phenomenon in the appropriate endocrine context can prevent unwarranted surgical intervention. In this patient, the presence of a pathogenic germline RAD51C c.404+2T>C variant appropriately prompted initiation of high-risk breast surveillance. Although RAD51C is associated with hereditary breast and ovarian cancer risk, it is not known to contribute to the pathogenesis of duct ectasia; its relevance in this case is primarily surveillance-related, providing the clinical context in which the lesion was detected.

## Conclusions

Mammary duct ectasia can mimic malignancy on MRI but is benign. In patients with relevant endocrine history, prolonged prolactin elevation may contribute to secretory stasis and ductal dilatation. Radiologic-pathologic correlation supports appropriate management and helps avoid overtreatment. To date, only one similar case linking pituitary pathology and mammary duct ectasia has been reported in the literature.
